# Crystal structure of diethyl 3-(3-chloro­phen­yl)-2,2-di­cyano­cyclo­propane-1,1-di­carboxyl­ate

**DOI:** 10.1107/S2056989016001444

**Published:** 2016-01-27

**Authors:** Nóra Veronika May, Gyula Tamás Gál, Zsolt Rapi, Péter Bakó

**Affiliations:** a Institute of Organic Chemistry, Research Centre for Natural Sciences, Hungarian Academy of Sciences, H-1519 Budapest, POB 206, Hungary; b Department of Organic Chemistry and Technology, Budapest University of Technology and Economics, H-1521 Budapest, POB 91, Hungary

**Keywords:** crystal structure, cyclo­propane derivatives, MIRC, phase-transfer catalysis, crown ether

## Abstract

In the racemic title compound, C_17_H_15_ClN_2_O_4_, which has been synthesized and the crystal structure of the solvent-free mol­ecule determined, the angle between the planes of the benzene and cyclo­propane rings is 54.29 (10)°. The mol­ecular conformation is stabilized by two weak intra­molecular C—H⋯O_carbox­yl_ inter­actions. In the crystal, C—H⋯O hydrogen bonds form centrosymmetric cyclic *R*_2_^2^(10) dimers which are linked into chain substructures extending along *c*. Further C—H⋯N_nitrile_ hydrogen bonding, including a centrosymmetric cyclic *R*_2_^2^(14) association, link the chain substructures, forming a two-dimensional layered structure extending across the approximate *ab* plane. No significant π–π or halogen–halogen inter­molecular inter­actions are present in the crystal.

## Abstract 2

The crystal structure of diethyl 3-(3-chloro­phen­yl)-2,2-di­cyano­cyclo­propane-1,1-di­carboxyl­ate shows one-dimensional chain substructures linked into two-dimensional layers of mol­ecules, through both *C*—*H*⋯O_carbox­yl_ and *C*—*H*⋯N_nitrile_ hydrogen bonds.

## Chemical context   

The formation of C—C bonds by the Michael addition of the appropriate carboanionic reagents to α,β-unsaturated car­bonyl compounds is one of the most useful methods of remote functionalization in organic synthesis (Mather *et al.*, 2006 ▸; Little *et al.*, 1995 ▸). The Michael Initiated Ring Closure (MIRC) reaction represents an elegant approach which has been applied extensively for the construction of cyclo­propane derivatives (Zheng *et al.*, 2005 ▸; Aggarwal & Grange, 2006 ▸). The cyclo­propane ring is an important building moiety for a large number of biologically active compounds and are subunits found in many natural products, so that the development of novel methods to provide new cyclo­propane derivatives is a challenge. The MIRC reaction strategy may also be utilized through a one-pot multicomponent reaction which has gained inter­est among synthetic organic chemists recently (Riches *et al.*, 2010 ▸). Many phase-transfer-catalyzed methods have been developed for the Michael reaction that are simple and environmentally friendly (Shioiri, 1997 ▸). We have developed a new phase-transfer-catalyzed method for the MIRC reaction that is both simple and environmentally friendly. The novel title compound, C_17_H_15_ClN_2_O_4_, was prepared in good yield in such a reaction using a sugar-based crown ether as the catalyst (Bakó *et al.*, 2015 ▸).
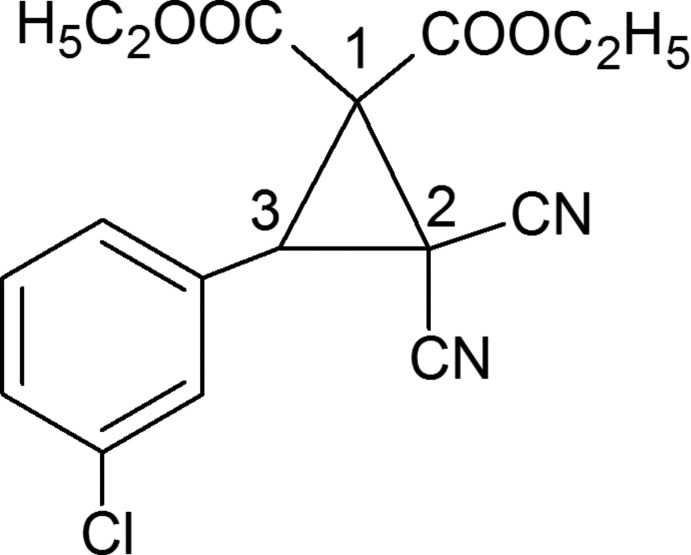


## Structural commentary   

**Figure 1:**
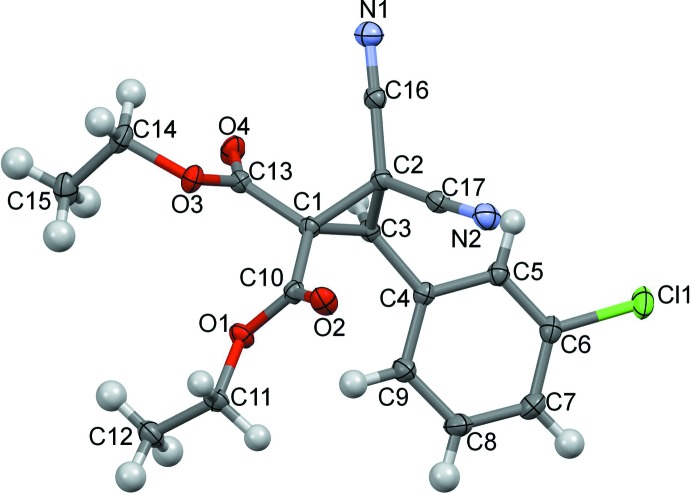
The mol­ecular structure of the title compound, showing the atom numbering. Displacement ellipsoids are drawn at the 50% probability level.

**Figure 2:**
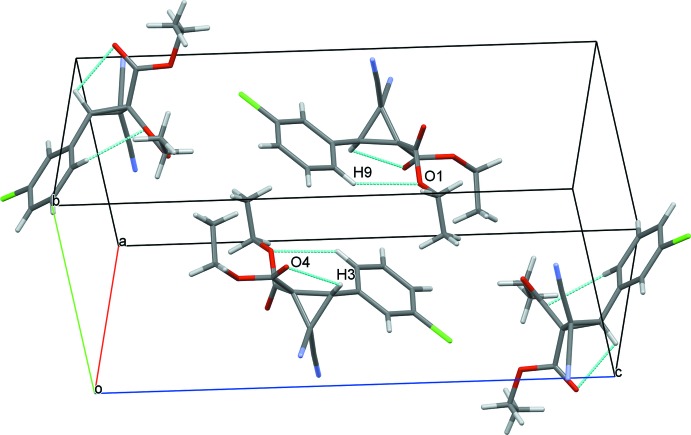
The four mol­ecules in the unit cell of the title compound, with the intra­molecular inter­actions shown as dashed lines.

In the mol­ecular structure of the title compound (Fig. 1 ▸), atom C3 is a chiral centre, but the racemic mixture crystallizes in the centrosymmetric space group *P*2_1_/*c*. The dihedral angle between the planes of the benzene and cyclo­propane rings is 54.29 (10)°, while the conformation is stabilized by two intra­molecular C—H⋯O_carbox­yl_ inter­actions, a weak C9—H⋯O1 hydrogen bond (Table 1 ▸) and a short intramolecular C3⋯O4 inter­action [2.8447 (16) Å] (Fig. 2 ▸).

## Supra­molecular features   

**Figure 3:**
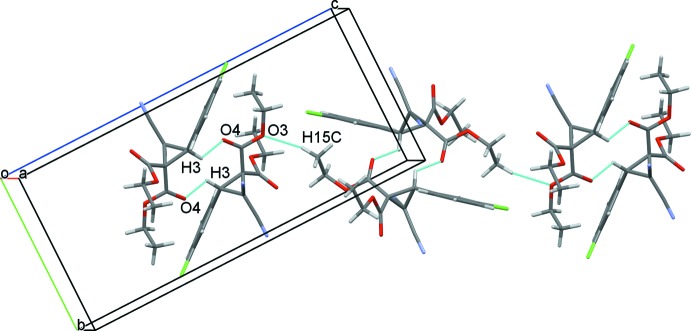
The one-dimensional chain polymer substructures in the title compound involving centrosymmetric cyclic C3—H⋯O4^i^ and C15–H⋯O3^ii^ hydrogen bonds (shown as dashed lines). For symmetry codes, see Table 1 ▸.

**Figure 4:**
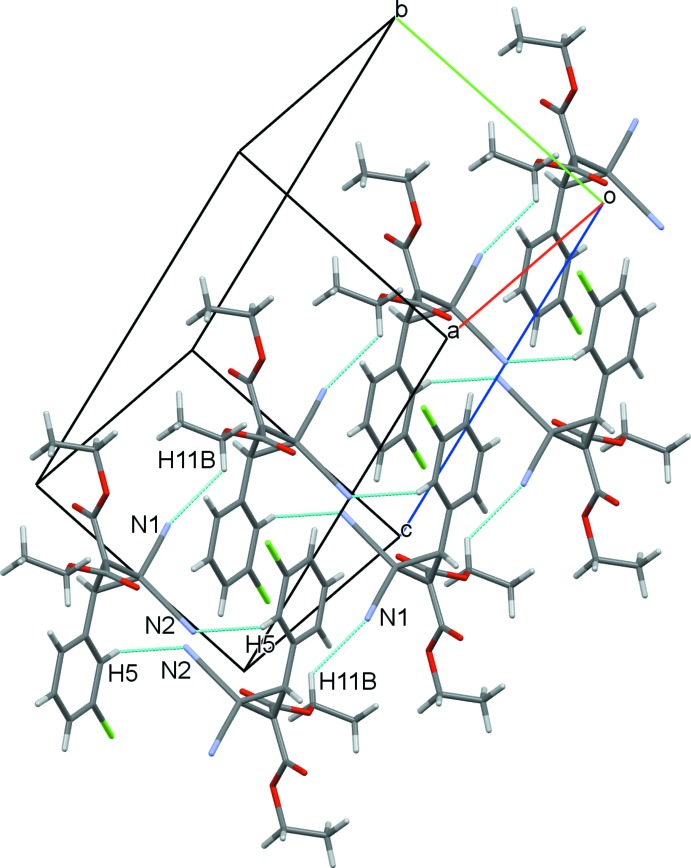
The two-dimensional sheet-like structure in the title compound, showing the centrosymmetric C5—H⋯N2^iii^ and C11—H⋯N1^iv^ hydrogen-bond extensions. For symmetry codes, see Table 1 ▸.

In the crystal, C3—H⋯O4^i^ hydrogen bonds (Table 1 ▸) form inversion dimers having a graph-set descriptor 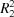
(10) (Bernstein *et al.*, 1995 ▸), and are linked into chain substructures extending along *c* through weak C15—H⋯O3^ii^ hydrogen bonds (Fig. 3 ▸). These chain substructures are further linked through centrosymmetric cyclic 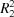
(14) C5—H⋯N2^iii^ and C11—H⋯N1^iv^ hydrogen-bonding inter­actions to nitrile N-atom acceptors, forming a two-dimensional layered structure extending across the approximate *ab* plane (Fig. 4 ▸). Although the mol­ecule contains an aromatic ring and a Cl atom, there are no significant π–π or halogen–halogen inter­actions in the crystal structure. The relatively high calculated density (1.383 Mg m^−3^) and the Kitaigorodskii packing index (KPI = 69.1) (Spek, 2009 ▸) show tight packing of the mol­ecules in the unit cell, which results in no residual solvent-accessible voids in the crystal.

## Database survey   

The crystal structure of many substituted phenyl­cyclo­propane derivatives have already been studied from which four closely related structures were chosen to compare the mol­ecular structures with the title compound. In the most relevant structures, the dihedral angle between the cyclo­propane and benzene rings was found to be very similar. For 1-cyano-3,3-dimethyl-*r*-2-*m*-nitro­phenyl-*t*-1-phenyl­cyclo­propane [Cam­bridge Structural Database (CSD; Groom & Allen, 2014 ▸) refcode GAHYOD; Tinant *et al.*, 1988 ▸], this value is 47.6°, for 2-(2,2-di­cyano­vin­yl)-*cis*-1,3-diphenyl-*cis*-1,2-diiso­propyl­cyclopropane (KANFOU; Zimmerman & Cassel, 1989 ▸) it is 50.8°, for diethyl 1,2-di­cyano-3-phenylcyclo­propane-1,2-di­carboxyl­ate (PEXFAZ; Elinson *et al.*, 1993 ▸) it is 48.0° and for (*E*)-trimethyl 2-cyano-3-phenyl­cyclo­propane-1,1,2-tri­carboxyl­ate (YEQSOC01; Elinson *et al.*, 2006 ▸) it is 49.2°. This suggests that although the benzene ring is capable of rotation about the C—C bond, the groups in close proximity on the other two cyclo­propane C atoms enforce this 47–53° angle between the planes of the cyclo­propane and benzene rings.

## Synthesis and crystallization   

The title compound was synthesized by the reaction of 2-(3-chloro­benzyl­idene)malono­nitrile with diethyl 2-bromo­mal­on­ate under phase-transfer conditions. The reaction was carried out in a solid/liquid two-phase system [Na_2_CO_3_/tetra­hydro­furan (THF)] in the presence of a gluco­pyran­oside-based crown ether as the catalyst. The compound was isolated by preparative thin-layer chromatography (TLC) (silica gel) in good yield (m.p. 355–357 K). The chemical structure of the compound was confirmed by ^1^H, ^13^C NMR and mass spectroscopies. The details of the synthesis were reported previously (Bakó *et al.*, 2015 ▸). Single crystals suitable for X-ray diffraction analysis were obtained by crystallization from ethanol.

## Refinement   

Crystal data, data collection and structure refinement details are summarized in Table 2 ▸. All H atoms were located in difference electron-density maps but were included in the structure refinement at calculated positions, with C—H = 0.95–1.00 Å, and allowed to ride, with *U*_iso_(H) = 1.2*U*_eq_(C).

## Supplementary Material

Crystal structure: contains datablock(s) I, header. DOI: 10.1107/S2056989016001444/zs2355sup1.cif

Structure factors: contains datablock(s) I. DOI: 10.1107/S2056989016001444/zs2355Isup2.hkl

Supporting information file. DOI: 10.1107/S2056989016001444/zs2355Isup3.cml

CCDC reference: 1449224

Additional supporting information:  crystallographic information; 3D view; checkCIF report

## Figures and Tables

**Table 1:** Hydrogen-bond geometry (Å, °)

*D*—H⋯*A*	*D*—H	H⋯*A*	*D*⋯*A*	*D*—H⋯*A*
C9—H9⋯O1	0.95	2.59	3.3529 (15)	138
C3—H3⋯O4^i^	1.00	2.45	3.1419 (16)	126
C15—H15*C*⋯O3^ii^	0.98	2.63	3.5656 (18)	161
C5—H5⋯N2^iii^	0.95	2.61	3.4621 (18)	150
C11—H11*B*⋯N1^iv^	0.99	2.63	3.3337 (17)	128

Symmetry codes: (i) 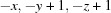; (ii) 
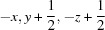
; (iii) 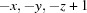; (iv) 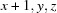.

**Table 2:** Experimental details

Crystal data	
Chemical formula	C_17_H_15_ClN_2_O_4_
*M*_r_	346.76
Crystal system, space group	Monoclinic, *P*2_1_/*c*
Temperature (K)	103
*a*, *b*, *c* (Å)	8.9221 (6), 9.1927 (7), 20.3446 (16)
β (°)	93.829 (2)
*V* (Å^3^)	1664.9 (2)
*Z*	4
Radiation type	Mo *K*α
μ (mm^−1^)	0.25
Crystal size (mm)	0.50 × 0.25 × 0.25

Data collection	
Diffractometer	R-AXIS RAPID
Absorption correction	empirical (*NUMABS*; Higashi, 2002 ▸)
*T*_min_, *T*_max_	0.755, 1.000
No. of measured, independent and observed [*I* > 2σ(*I*)] reflections	57969, 5052, 4312
*R*_int_	0.042
(sin θ/λ)_max_ (Å^−1^)	0.714

Refinement	
*R*[*F* ^2^ > 2σ(*F* ^2^)], *wR*(*F* ^2^), *S*	0.042, 0.113, 1.11
No. of reflections	5052
No. of parameters	219
H-atom treatment	H-atom parameters constrained
Δρ_max_, Δρ_min_ (e Å^−3^)	0.49, −0.31

Computer programs: *CrystalClear* (Rigaku/MSC, 2008 ▸), *SHELXS97* (Sheldrick, 2008 ▸), *SHELXL2014* (Sheldrick, 2015 ▸) and *Mercury* (Macrae *et al.*, 2006 ▸).

## supplementary crystallographic information

### Crystal data

**Table:**

C_17_H_15_ClN_2_O_4_	*D*_x_ = 1.383 Mg m^−^^3^
*M**_r_* = 346.76	Melting point = 355–357 K
Monoclinic, *P*2_1_/*c*	Mo *K*α radiation, λ = 0.71073 Å
*a* = 8.9221 (6) Å	Cell parameters from 37218 reflections
*b* = 9.1927 (7) Å	θ = 3.0–30.5°
*c* = 20.3446 (16) Å	µ = 0.25 mm^−^^1^
β = 93.829 (2)°	*T* = 103 K
*V* = 1664.9 (2) Å^3^	Block, colorless
*Z* = 4	0.50 × 0.25 × 0.25 mm
*F*(000) = 720	

### Data collection

**Table:**

RAXIS-RAPID diffractometer	5052 independent reflections
Radiation source: sealed tube	4312 reflections with *I* > 2σ(*I*)
Graphite monochromator	*R*_int_ = 0.042
Detector resolution: 10.0000 pixels mm^-1^	θ_max_ = 30.5°, θ_min_ = 3.0°
dtprofit.ref scans	*h* = −12→12
Absorption correction: empirical (using intensity measurements) (*NUMABS*; Higashi, 2002)	*k* = −13→13
*T*_min_ = 0.755, *T*_max_ = 1.000	*l* = −29→29
57969 measured reflections	

### Refinement

**Table:**

Refinement on *F*^2^	Primary atom site location: difference Fourier map
Least-squares matrix: full	Secondary atom site location: difference Fourier map
*R*[*F*^2^ > 2σ(*F*^2^)] = 0.042	Hydrogen site location: difference Fourier map
*wR*(*F*^2^) = 0.113	H-atom parameters constrained
*S* = 1.11	*w* = 1/[σ^2^(*F*_o_^2^) + (0.0475*P*)^2^ + 0.9362*P*] where *P* = (*F*_o_^2^ + 2*F*_c_^2^)/3
5052 reflections	(Δ/σ)_max_ = 0.001
219 parameters	Δρ_max_ = 0.49 e Å^−^^3^
0 restraints	Δρ_min_ = −0.31 e Å^−^^3^

### Special details

**Table:**

Geometry. All e.s.d.'s (except the e.s.d. in the dihedral angle between two l.s. planes) are estimated using the full covariance matrix. The cell e.s.d.'s are taken into account individually in the estimation of e.s.d.'s in distances, angles and torsion angles; correlations between e.s.d.'s in cell parameters are only used when they are defined by crystal symmetry. An approximate (isotropic) treatment of cell e.s.d.'s is used for estimating e.s.d.'s involving l.s. planes.

### Fractional atomic coordinates and isotropic or equivalent isotropic displacement parameters (Å^2^)

**Table:**

	*x*	*y*	*z*	*U*_iso_*/*U*_eq_	
Cl1	0.28965 (4)	−0.00643 (4)	0.66880 (2)	0.02905 (10)	
O3	0.08826 (11)	0.51460 (10)	0.31362 (4)	0.02054 (19)	
O1	0.39223 (10)	0.41867 (10)	0.35507 (4)	0.01960 (19)	
O2	0.29321 (11)	0.19550 (11)	0.33457 (5)	0.0237 (2)	
O4	0.04757 (12)	0.59834 (11)	0.41513 (5)	0.0240 (2)	
N1	−0.21756 (13)	0.33382 (14)	0.43288 (6)	0.0254 (2)	
N2	0.10162 (14)	−0.01960 (13)	0.42424 (6)	0.0250 (2)	
C13	0.09652 (14)	0.50931 (13)	0.37904 (6)	0.0167 (2)	
C16	−0.09432 (14)	0.29951 (14)	0.43266 (6)	0.0181 (2)	
C3	0.17943 (13)	0.34622 (13)	0.47595 (6)	0.0156 (2)	
H3	0.1350	0.4276	0.5008	0.019*	
C5	0.24907 (14)	0.17626 (14)	0.56603 (6)	0.0180 (2)	
H5	0.1453	0.1708	0.5737	0.022*	
C1	0.17187 (13)	0.36875 (13)	0.40292 (6)	0.0153 (2)	
C6	0.35355 (15)	0.09894 (14)	0.60500 (6)	0.0203 (2)	
C10	0.29152 (14)	0.31323 (14)	0.35989 (6)	0.0167 (2)	
C17	0.08762 (14)	0.10373 (14)	0.42559 (6)	0.0188 (2)	
C2	0.06331 (14)	0.25936 (13)	0.43230 (6)	0.0163 (2)	
C9	0.45070 (15)	0.27250 (16)	0.50559 (6)	0.0224 (3)	
H9	0.4847	0.3334	0.4719	0.027*	
C8	0.55346 (15)	0.19268 (17)	0.54558 (7)	0.0267 (3)	
H8	0.6575	0.1988	0.5386	0.032*	
C7	0.50616 (16)	0.10434 (16)	0.59547 (7)	0.0248 (3)	
H7	0.5762	0.0491	0.6224	0.030*	
C11	0.51527 (14)	0.38886 (15)	0.31296 (6)	0.0201 (2)	
H11A	0.4754	0.3568	0.2687	0.024*	
H11B	0.5814	0.3116	0.3325	0.024*	
C14	0.02312 (17)	0.64730 (16)	0.28356 (7)	0.0253 (3)	
H14A	−0.0618	0.6804	0.3087	0.030*	
H14B	−0.0161	0.6263	0.2379	0.030*	
C12	0.60066 (16)	0.52914 (16)	0.30825 (7)	0.0265 (3)	
H12B	0.5329	0.6052	0.2901	0.032*	
H12C	0.6831	0.5156	0.2793	0.032*	
H12A	0.6416	0.5580	0.3522	0.032*	
C15	0.13952 (19)	0.76533 (16)	0.28303 (7)	0.0289 (3)	
H15C	0.0970	0.8502	0.2593	0.035*	
H15B	0.2268	0.7298	0.2610	0.035*	
H15A	0.1707	0.7930	0.3284	0.035*	
C4	0.29805 (14)	0.26271 (13)	0.51514 (6)	0.0166 (2)	

### Atomic displacement parameters (Å^2^)

**Table:**

	*U*^11^	*U*^22^	*U*^33^	*U*^12^	*U*^13^	*U*^23^
Cl1	0.0331 (2)	0.02836 (18)	0.02491 (17)	−0.00564 (13)	−0.00412 (13)	0.01159 (12)
O3	0.0264 (5)	0.0190 (4)	0.0161 (4)	0.0027 (4)	−0.0001 (3)	0.0024 (3)
O1	0.0199 (4)	0.0187 (4)	0.0210 (4)	−0.0014 (3)	0.0072 (3)	−0.0024 (3)
O2	0.0277 (5)	0.0187 (4)	0.0256 (5)	−0.0004 (4)	0.0096 (4)	−0.0045 (4)
O4	0.0312 (5)	0.0201 (5)	0.0215 (4)	0.0085 (4)	0.0073 (4)	0.0017 (4)
N1	0.0210 (6)	0.0288 (6)	0.0263 (6)	0.0004 (5)	0.0012 (4)	−0.0003 (5)
N2	0.0228 (6)	0.0193 (5)	0.0331 (6)	−0.0023 (4)	0.0035 (5)	−0.0016 (4)
C13	0.0158 (5)	0.0169 (5)	0.0176 (5)	−0.0002 (4)	0.0017 (4)	0.0017 (4)
C16	0.0181 (6)	0.0186 (6)	0.0176 (5)	−0.0012 (4)	0.0011 (4)	0.0002 (4)
C3	0.0174 (5)	0.0144 (5)	0.0153 (5)	−0.0003 (4)	0.0031 (4)	−0.0004 (4)
C5	0.0192 (6)	0.0166 (5)	0.0182 (5)	−0.0011 (4)	0.0007 (4)	0.0004 (4)
C1	0.0162 (5)	0.0144 (5)	0.0155 (5)	0.0005 (4)	0.0030 (4)	0.0002 (4)
C6	0.0252 (6)	0.0174 (6)	0.0179 (5)	−0.0014 (5)	−0.0018 (4)	0.0022 (4)
C10	0.0189 (5)	0.0167 (5)	0.0145 (5)	0.0019 (4)	0.0029 (4)	0.0015 (4)
C17	0.0167 (6)	0.0193 (6)	0.0205 (5)	−0.0022 (4)	0.0021 (4)	−0.0002 (4)
C2	0.0160 (5)	0.0154 (5)	0.0175 (5)	−0.0004 (4)	0.0017 (4)	0.0002 (4)
C9	0.0189 (6)	0.0274 (7)	0.0209 (6)	0.0002 (5)	0.0024 (4)	0.0040 (5)
C8	0.0173 (6)	0.0349 (8)	0.0279 (6)	0.0032 (5)	0.0009 (5)	0.0041 (6)
C7	0.0242 (7)	0.0252 (7)	0.0244 (6)	0.0048 (5)	−0.0031 (5)	0.0026 (5)
C11	0.0188 (6)	0.0230 (6)	0.0194 (5)	0.0012 (5)	0.0065 (4)	0.0002 (5)
C14	0.0299 (7)	0.0228 (6)	0.0227 (6)	0.0058 (5)	−0.0016 (5)	0.0068 (5)
C12	0.0241 (7)	0.0260 (7)	0.0302 (7)	−0.0034 (5)	0.0083 (5)	0.0022 (5)
C15	0.0395 (8)	0.0199 (6)	0.0281 (7)	0.0019 (6)	0.0079 (6)	0.0031 (5)
C4	0.0189 (6)	0.0154 (5)	0.0155 (5)	0.0008 (4)	0.0007 (4)	−0.0003 (4)

### Geometric parameters (Å, º)

**Table:**

Cl1—C6	1.7455 (13)	C6—C7	1.389 (2)
O1—C10	1.3298 (16)	C7—C8	1.387 (2)
O1—C11	1.4628 (15)	C8—C9	1.393 (2)
O2—C10	1.1991 (16)	C11—C12	1.504 (2)
O3—C13	1.3290 (15)	C14—C15	1.503 (2)
O3—C14	1.4671 (17)	C3—H3	1.0000
O4—C13	1.2010 (16)	C5—H5	0.9500
N1—C16	1.1442 (17)	C7—H7	0.9500
N2—C17	1.1411 (18)	C8—H8	0.9500
C1—C2	1.5440 (17)	C9—H9	0.9500
C1—C3	1.4972 (17)	C11—H11A	0.9900
C1—C10	1.5137 (17)	C11—H11B	0.9900
C1—C13	1.5212 (17)	C12—H12A	0.9800
C2—C3	1.5417 (17)	C12—H12B	0.9800
C2—C16	1.4545 (18)	C12—H12C	0.9800
C2—C17	1.4548 (18)	C14—H14A	0.9900
C3—C4	1.4941 (17)	C14—H14B	0.9900
C4—C5	1.3982 (17)	C15—H15A	0.9800
C4—C9	1.3915 (18)	C15—H15B	0.9800
C5—C6	1.3799 (18)	C15—H15C	0.9800
			
C10—O1—C11	116.35 (10)	N1—C16—C2	178.70 (14)
C13—O3—C14	116.22 (10)	N2—C17—C2	175.24 (14)
C2—C1—C3	60.90 (8)	C1—C3—H3	114.00
C2—C1—C10	119.29 (10)	C2—C3—H3	114.00
C2—C1—C13	113.68 (10)	C4—C3—H3	114.00
C3—C1—C10	122.72 (10)	C4—C5—H5	121.00
C3—C1—C13	115.09 (10)	C6—C5—H5	120.00
C10—C1—C13	114.55 (10)	C6—C7—H7	121.00
C1—C2—C3	58.05 (8)	C8—C7—H7	121.00
C1—C2—C16	117.93 (10)	C7—C8—H8	119.00
C1—C2—C17	120.19 (11)	C9—C8—H8	120.00
C3—C2—C16	118.59 (10)	C4—C9—H9	120.00
C3—C2—C17	117.69 (10)	C8—C9—H9	120.00
C16—C2—C17	113.60 (11)	O1—C11—H11A	110.00
C1—C3—C2	61.05 (8)	O1—C11—H11B	110.00
C1—C3—C4	125.73 (10)	C12—C11—H11A	110.00
C2—C3—C4	117.78 (10)	C12—C11—H11B	110.00
C3—C4—C5	116.23 (11)	H11A—C11—H11B	109.00
C3—C4—C9	123.87 (11)	C11—C12—H12A	109.00
C5—C4—C9	119.83 (11)	C11—C12—H12B	109.00
C4—C5—C6	119.06 (12)	C11—C12—H12C	109.00
Cl1—C6—C5	118.13 (10)	H12A—C12—H12B	109.00
Cl1—C6—C7	119.71 (10)	H12A—C12—H12C	109.00
C5—C6—C7	122.16 (12)	H12B—C12—H12C	109.00
C6—C7—C8	118.16 (13)	O3—C14—H14A	110.00
C7—C8—C9	121.00 (13)	O3—C14—H14B	110.00
C4—C9—C8	119.77 (12)	C15—C14—H14A	110.00
O1—C10—O2	126.75 (12)	C15—C14—H14B	110.00
O1—C10—C1	107.64 (10)	H14A—C14—H14B	108.00
O2—C10—C1	125.62 (12)	C14—C15—H15A	109.00
O1—C11—C12	106.25 (11)	C14—C15—H15B	109.00
O3—C13—O4	126.10 (12)	C14—C15—H15C	109.00
O3—C13—C1	110.16 (10)	H15A—C15—H15B	109.00
O4—C13—C1	123.68 (11)	H15A—C15—H15C	109.00
O3—C14—C15	110.41 (12)	H15B—C15—H15C	109.00
			
C10—O1—C11—C12	172.57 (10)	C3—C1—C10—O2	88.80 (16)
C11—O1—C10—C1	−177.59 (9)	C13—C1—C10—O1	56.21 (13)
C11—O1—C10—O2	1.86 (18)	C13—C1—C10—O2	−123.25 (14)
C14—O3—C13—C1	−177.70 (10)	C3—C1—C10—O1	−91.74 (13)
C14—O3—C13—O4	4.90 (19)	C2—C1—C10—O1	−164.13 (10)
C13—O3—C14—C15	82.31 (14)	C2—C1—C10—O2	16.41 (19)
C13—C1—C2—C3	−106.61 (11)	C17—C2—C3—C1	110.00 (12)
C10—C1—C2—C17	7.68 (17)	C1—C2—C3—C4	−117.58 (12)
C2—C1—C3—C4	104.97 (13)	C16—C2—C3—C1	−106.79 (12)
C10—C1—C3—C2	−107.93 (13)	C16—C2—C3—C4	135.63 (12)
C10—C1—C3—C4	−2.96 (18)	C17—C2—C3—C4	−7.58 (16)
C13—C1—C3—C2	104.29 (11)	C1—C3—C4—C5	−140.24 (12)
C13—C1—C3—C4	−150.75 (11)	C1—C3—C4—C9	42.95 (19)
C13—C1—C2—C16	1.30 (15)	C2—C3—C4—C9	115.80 (14)
C13—C1—C2—C17	147.67 (11)	C2—C3—C4—C5	−67.40 (15)
C3—C1—C2—C16	107.92 (12)	C3—C4—C9—C8	178.59 (12)
C3—C1—C2—C17	−105.72 (12)	C9—C4—C5—C6	−1.63 (19)
C10—C1—C2—C3	113.39 (12)	C3—C4—C5—C6	−178.57 (11)
C10—C1—C2—C16	−138.69 (12)	C5—C4—C9—C8	1.9 (2)
C2—C1—C13—O3	−108.93 (12)	C4—C5—C6—C7	0.2 (2)
C2—C1—C13—O4	68.55 (16)	C4—C5—C6—Cl1	179.25 (10)
C3—C1—C13—O3	−176.52 (10)	Cl1—C6—C7—C8	−178.06 (11)
C3—C1—C13—O4	0.96 (18)	C5—C6—C7—C8	1.0 (2)
C10—C1—C13—O3	33.02 (14)	C6—C7—C8—C9	−0.8 (2)
C10—C1—C13—O4	−149.50 (13)	C7—C8—C9—C4	−0.7 (2)

### Hydrogen-bond geometry (Å, º)

**Table:**

*D*—H···*A*	*D*—H	H···*A*	*D*···*A*	*D*—H···*A*
C3—H3···O4	1.00	2.43	2.8447 (16)	104
C9—H9···O1	0.95	2.59	3.3529 (15)	138
C3—H3···O4^i^	1.00	2.45	3.1419 (16)	126
C15—H15*C*···O3^ii^	0.98	2.63	3.5656 (18)	161
C5—H5···N2^iii^	0.95	2.61	3.4621 (18)	150
C11—H11*B*···N1^iv^	0.99	2.63	3.3337 (17)	128

Symmetry codes: (i) −*x*, −*y*+1, −*z*+1; (ii) −*x*, *y*+1/2, −*z*+1/2; (iii) −*x*, −*y*, −*z*+1; (iv) *x*+1, *y*, *z*.
